# Cell-based assays and comparative genomics revealed the conserved and hidden effects of *Wolbachia* on insect sex determination

**DOI:** 10.1093/pnasnexus/pgae348

**Published:** 2024-08-22

**Authors:** Hiroshi Arai, Benjamin Herran, Takafumi N Sugimoto, Mai Miyata, Tetsuhiko Sasaki, Daisuke Kageyama

**Affiliations:** Institute of Agrobiological Sciences, National Agriculture and Food Research Organization (NARO), Ibaraki 305-0851, Japan; United Graduate School of Agricultural Science, Tokyo University of Agriculture and Technology, Tokyo 183-8509, Japan; Institute of Agrobiological Sciences, National Agriculture and Food Research Organization (NARO), Ibaraki 305-0851, Japan; Institute of Agrobiological Sciences, National Agriculture and Food Research Organization (NARO), Ibaraki 305-0851, Japan; Faculty of Engineering, University of Fukui, Fukui 910-8507, Japan; Graduate School of Agriculture, Honeybee Science Research Center, Research Institute, Tamagawa University, Tokyo 194-8610, Japan; Institute of Agrobiological Sciences, National Agriculture and Food Research Organization (NARO), Ibaraki 305-0851, Japan

**Keywords:** cell culture, endosymbiosis, male killing, Oscar, *Wolbachia*

## Abstract

It is advantageous for maternally transmitted endosymbionts to skew the sex ratio of their hosts toward females. Some endosymbiotic bacteria, such as *Wolbachia*, cause their insect hosts to exclusively produce female offspring through male killing (MK) or feminization. In some lepidopteran insects, MK is achieved by affecting the sex-determining process in males, and a unique mechanism of MK and its functional link with feminization have been implicated. However, comparative analysis of these phenotypes is often difficult because they have been analyzed in different host–symbiont systems, and transinfection of *Wolbachia* across different hosts is often challenging. In this study, we demonstrated the effects of nine *Wolbachia* strains on the splicing of sex-determining genes in Lepidoptera by fixing the host genetic background using a cell culture system. Cell transinfection assays confirmed that three MK-inducing *Wolbachia* strains and one feminization-inducing *Wolbachia* strain increased the female-type splicing products of the core sex-determining genes *doublesex*, *masculinizer*, and *zinc finger protein 2*. Regarding *Wolbachia* strains that do not induce MK/feminization, three had no effect on these sex-determining genes, whereas two strains induced female-type splicing of *masculinizer* and *doublesex* but not *zinc finger protein 2*. Comparative genomics confirmed that homologs of *oscar*, the *Wolbachia* gene responsible for MK in *Ostrinia*, were encoded by four MK/feminizing *Wolbachia* strains, but not by five non-MK/nonfeminizing strains. These results support the conserved effects underlying MK and feminization induced by *oscar*-bearing *Wolbachia* and suggested other potential mechanisms that *Wolbachia* might employ to manipulate host sex.

**Significance Statement**

Arthropods often carry maternally transmitted symbionts, such as *Wolbachia*. The lack of paternal transmission frequently led them to evolve host manipulating behaviors in the favor of female hosts, substantiated by male killing and feminization. Although *Wolbachia* induces these phenotypes in a wide range of insects, the underlying mechanisms, diversity, and commonality remain largely unclear. In this study, a combination of transinfection assays and comparative genomics revealed the conserved effects of male killing and feminizing *Wolbachia* strains on lepidopteran sex determination. Furthermore, some nonmale killing/nonfeminizing *Wolbachia* strains were also shown to have an inherent ability to influence sex determination, albeit in a different manner, suggesting the potential for multiple mechanisms to manipulate host sex.

## Introduction

Most animal species have distinct male and female forms that are determined by their genomic information during early development in a process called sex determination (1, 2). However, in many cases, sex-determining systems are manipulated or overridden by maternally transmitted cytoplasmic elements (3–6). In arthropods, several bacterial endosymbionts manipulate host reproduction to produce only female progenies (parthenogenesis) and by killing (i.e. male killing [MK]) or feminizing males (feminization [FM]) (7–11). The maternally inherited bacterium *Wolbachia* (Alphaproteobacteria) is a genus of typical microbes that manipulate host reproduction through various mechanisms (12). For example, *Wolbachia* species induce parthenogenesis, MK, and FM in diverse insects belonging to phylogenetically distinct taxa (e.g. Diptera, Lepidoptera, Hymenoptera, and Coleoptera) and other arthropods (8, 9, 13–16). *Wolbachia* species are estimated to inhabit at least 40% of all insect species, making them among the most widespread endosymbionts globally (12, 17).

It has been hypothesized that the mechanisms underlying MK and FM are associated with sex-determination cascades in insects (18). Indeed, some MK *Wolbachia* strains directly interact with sex-determination systems in lepidopteran insects (19–22). The lepidopteran sex-determination system consists of multiple transcriptional regulators, some of which exhibit sex-linked expression and/or splicing isoforms. Lepidopterans generally have a female heterogametic sex chromosome system (e.g. WZ/ZZ) and employ the dosage compensation system, which equalizes the sex-linked (Z-chromosome-linked) gene dose between males and females (20, 21). The dosage compensation system is regulated by a Z-linked CCCH-type zinc finger motif encoding gene, *masculinizer* (*masc*), which is critical for development as a male. The *masc* gene also regulates the downstream master regulator of sex determination and differentiation, *doublesex* (*dsx*), which exhibits sex-dependent splicing isoforms (*dsxF* in females and *dsxM* in males) (23, 24). *Ostrinia* and *Homona* male moths use these factors in their sex-determination cascades, and MK *Wolbachia* strains disrupt the expression of the *masc* gene and induce the “female” isoform of *dsx* (*dsxF*), leading to a “mismatch” between genetic sex (male: ZZ sex chromosome constitution) and phenotypic sex (female: based on *dsxF*) and male death (19–22). In addition to *dsx*, *masc* also exhibits splicing variation in the untranslated region between males and females of *Ostrinia* and *Homona* moths, and MK *Wolbachia* suppresses the expression levels of the male-type *masc* isoforms in male embryos (22, 25). Although the extent to which it is involved in sex-determination remains unclear, *Ostrinia* moths exhibit male- and female-type splicing variants of the autosomal CCCH-type zinc finger motif encoding gene *zinc finger protein 2* (*znf2*) (25), which is an upstream regulator of *dsx* in the silkworm *Bombyx mori* (26). In male embryos, MK *Wolbachia* (*w*Sca) induces a female-specific transcript variant and reduces the male-type isoforms of *znf2*. Similarly, the FM *Wolbachia* strain *w*Fem induces *dsxF* production in *Eurema* butterflies (27).

More recently, a *Wolbachia* protein Oscar (abbreviation of “Osugoroshi [i.e. MK, in Japanese] protein containing CifB C-terminus-like domain and many Ankyrin Repeats”) was shown to recapitulate the *Wolbachia*-induced MK phenotype in *Ostrinia* (23). The Oscar protein degrades and interacts Masc, which leads to the failure of dosage compensation and the production of female-type *dsx* isoforms in *Ostrinia* male moths (23, 24). Furthermore, an Oscar homolog was found in the prophage region of an MK *Wolbachia* strain in *Homona* moths (28). These findings imply a general role for Oscar in MK in lepidopteran insects, which have not been demonstrated. Based on the *Wolbachia* strains sequenced to date, Oscar is not widely conserved among MK *Wolbachia* strains (28). In addition, transgenically overexpressed Oscar does not function in *Drosophila* flies, which do not employ *masc* in sex determination. Instead, another *Wolbachia* factor, Wmk, induces male lethality (28, 29). In contrast to Oscar, Wmk homologs are widely conserved among *Wolbachia* strains regardless of their phenotypes (28, 29). These findings imply that *Wolbachia* uses multiple mechanisms to induce MK in insects, but their similarity/diversity and functional link with FM remain elusive.

The induction of *Wolbachia*-induced reproductive manipulation is deeply associated with the host genetic background (30–33). MK and FM can significantly distort the sex ratio of the host population toward females (13, 31, 34–36). Under extremely female-biased sex ratios, male production is strongly selected, and host suppressor mutations have evolved to mask the endosymbiont-induced MK in some insects, such as butterflies, ladybugs, lacewings, and planthoppers (36–40). Therefore, the absence of obvious *Wolbachia*-induced phenotypes in the natural host does not necessarily indicate the absence of *Wolbachia* MK capabilities. Thus, investigations of the inherent MK/FM abilities of *Wolbachia* strains are warranted to elucidate the genetic basis of these phenotypes.

In this study, we reveal a conserved and hidden feminizing effect induced by *Wolbachia* strains in Lepidoptera. Transinfection assays using a recently established cell culture system (25) revealed that MK/FM *Wolbachia* strains altered the splicing patterns of genes involved in the moth sex-determination cascade. Genomic analyses confirmed the presence of Oscar in MK/FM *Wolbachia* strains. Furthermore, an inherent feminizing ability was observed in Oscar-deficient *Wolbachia* strains that do not induce MK or FM in their native hosts. Based on these findings, we discuss the underlying mechanisms of MK and FM in Lepidoptera and highlight the diversity of causative factors.

## Results

### Transinfection assays revealed the feminizing abilities of *Wolbachia* strains in *Ostrinia* male cells

**Fig. 1.**
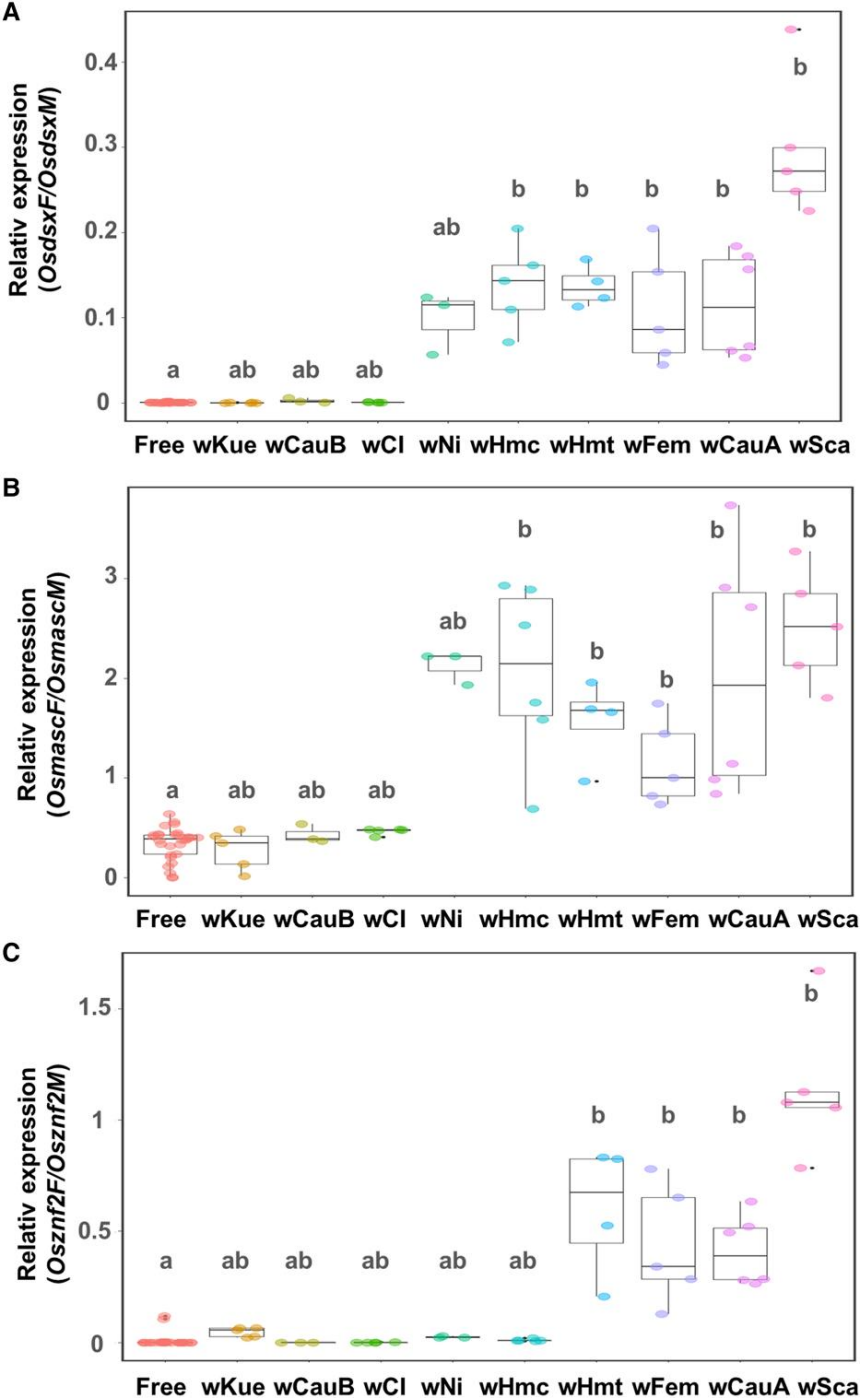
Feminizing effects of *Wolbachia* strains on *Ostrinia* male cells. Relative expression of *OsdsxF/OsdsxM* (A), *OsmascF/OsmascM* (B), and *Osznf2F/Osznf2M* (C) was quantified in *Wolbachia*-transinfected and control cells. Different letters above the whisker plots indicate significant differences based on the Steel–Dwass test (*P* < 0.05). Dot plots indicate replicates.

The NARO-Ossc-M1 (OsM1) cell line derived from a male moth (*Ostrinia scapulalis*) (25) was transinfected with the native *Wolbachia* strain *w*Sca and eight nonnative *Wolbachia* strains from lepidopteran insects (Table 1). Consistent with previous findings (25), transinfection with *w*Sca (native male killer) induced female-type splicing patterns in *dsx* (*OsdsxF*), *masc* (*OsmascF*), and *znf2* (*Osznf2F*) at 6 weeks posttransinfection (6 wpt) compared with the findings in nontransinfected controls (Steel–Dwass test; *Osdsx*: *P* = 0.02; *Osmasc*: *P* = 0.02; *Osznf2*: *P* = 0.02; Fig. 1). Albeit less or more conspicuously, *w*CauA, *w*Hm-t, and *w*Fem, having MK/FM abilities in native or nonnative hosts, also increased the expression of *OsdsxF* (*w*CauA: *P* = 0.01, *w*Hm-t: *P* = 0.04, *w*Fem: *P* = 0.02), *OsmascF* (*w*CauA: *P* = 0.01, *w*Hm-t: *P* = 0.04, *w*Fem: *P* = 0.02), and *Osznf2F* (*w*CauA: *P* = 0.01, *w*Hm-t: *P* = 0.04, *w*Fem: *P* = 0.02) at 6 wpt compared with the control levels. In contrast, cells transinfected with either *w*Kue, *w*CauB, or *w*CI (inducing cytoplasmic incompatibility but not MK/FM in native hosts) continued to exhibit male-type splicing patterns for *dsx* (*OsdsxM*), (*w*Kue: *P* = 0.48, *w*CauB: *P* = 0.99, *w*CI: *P* = 0.99), *masc* (*OsmascM*) (*w*Kue: *P* = 0.99, *w*CauB: *P* = 0.99, *w*CI: *P* = 0.44), and *znf2* (*Osznf2M*) (*w*Kue: *P* = 0.07, *w*CauB: *P* = 0.13, *w*CI: *P* = 0.80) similarly as observed in *Wolbachia*-free control cells. Unexpectedly, *w*Hm-c, which does not induce MK/FM in its host (43, 44), up-regulated *OsdsxF* (*P* = 0.02) and *OsmascF* (*P* = 0.02), but not *Osznf2F* (*P* = 0.07), at 6 wpt compared with the control levels. Similar patterns were also observed for the non-MK/FM *w*Ni1 strain in *Trichoplusia ni*, including the upregulation of *OsdsxF* (*P* = 0.13) and *OsmascF* (*P* = 0.13), albeit without significance, whereas no effect was observed on *Osznf2F* (*P* = 0.31; Fig. 1).

**Table 1.** Characteristics of the *Wolbachia* strains used in this study.

Strain	*w*Kue	*w*CauA	*w*CauB	*w*Sca	*w*CI	*w*CI + *w*Fem	*w*Ni1	*w*Hm-t	*w*Hm-c
Phenotype	CI	CI, MK^a^	CI	MK	CI	FM^b^	Non-MK, Non-FM	MK	Non-MK, Non-CI
Host	*E. kuehniella*	*C. cautella*	*C. cautella*	*O. scapulalis*	*Eu. mandarina*	*Eu. mandarina*	*T. ni*	*H. magnanima*	*H. magnanima*
Reference	Sasaki et al. (30)	Sasaki et al. (30)	Sasaki et al. (30)	Sugimoto and Ishikawa (19)	Hiroki et al. (41)	Hiroki et al. (41)	Miyata (pers. obs.)	Arai et al. (42)	Arai et al. (43)

MK, male killing; NMK, nonmale killing; Fem, feminization; CI, cytoplasmic incompatibility.

^a^ *w*CauA induces CI in its native host *C. cautella*, but MK in *E. kuehniella* (30).

^b^ *w*Fem always coexists with *w*CI.

### Oscar is conserved among MK and FM *Wolbachia* strains in Lepidoptera

**Fig. 2.**
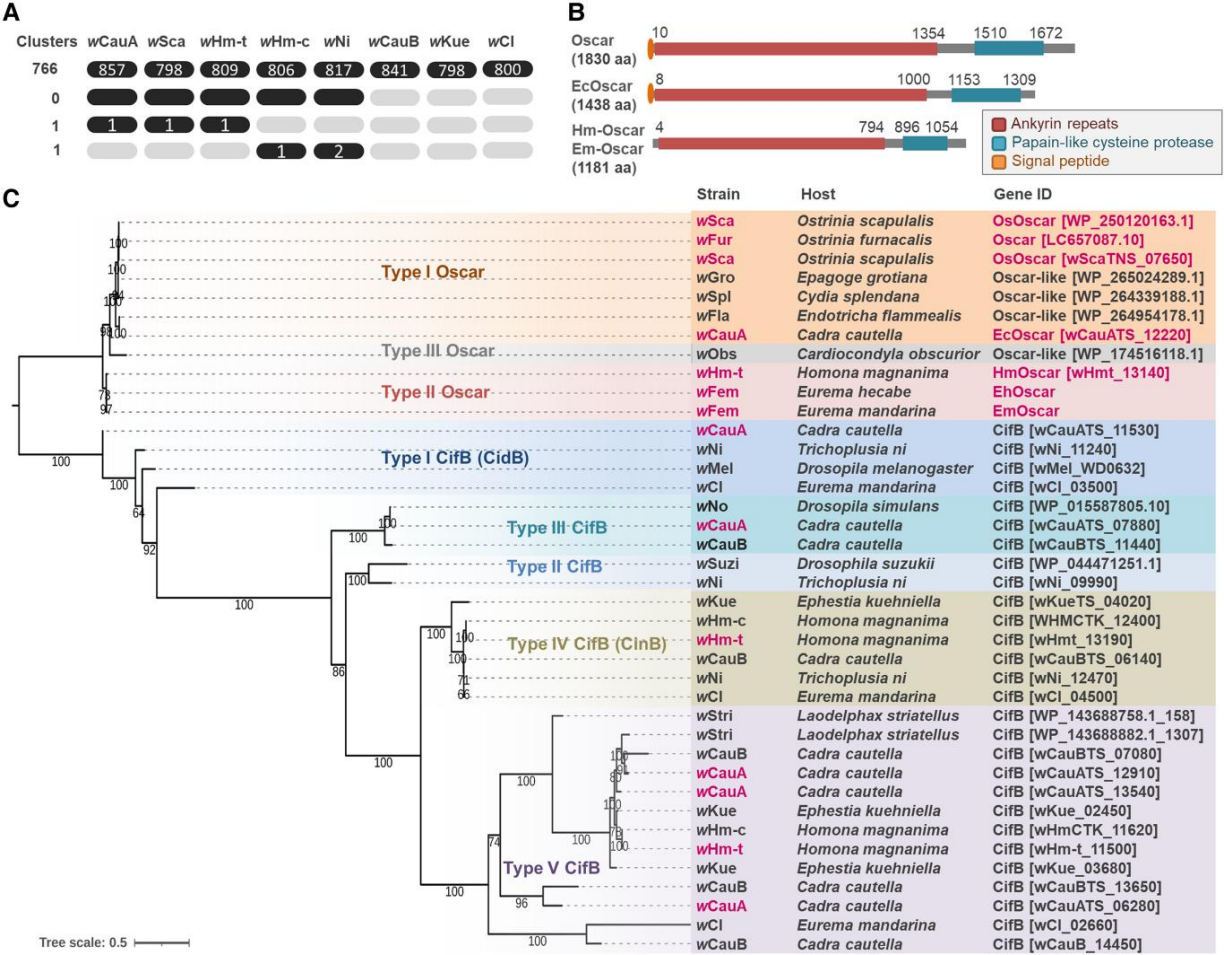
Phylogenies and structures of the Oscar and Cif proteins. A) Protein clusters conserved among the *Wolbachia* strains used in this study. The number of clusters is shown on the left, and the number of proteins contained in the clusters is indicated by black ellipses. B) Structure of Oscar and its homologs. aa, amino acids. The structure of Oscar (1,830 aa), derived from the MK *w*Fur strain, is based on Katsuma et al. (23). C) Phylogeny of Oscar and CifB homologs. Accession and gene numbers are given in parentheses.

A series of genome assembly and polishing processes were used to construct closed genomes of the *Wolbachia* strains *w*Sca, *w*CauA, *w*CauB, *w*Kue, *w*CI, *w*Ni1, and *w*Hm-c, which were used for the transactivation assays (Table 2). Comparative genomics using seven newly sequenced strains and the *w*Hm-t strain (28) identified 788 protein clusters shared by the *Wolbachia* strains, including Cifs (CifB and CifA) and Wmk homologs (Fig. 2A and Table S1). Oscar homologs were the only protein clusters shared by MK *Wolbachia* (*w*Sca, *w*CauA, and *w*Hm-t) but are absent in non-MK *Wolbachia* strains (*w*CauB, *w*Kue, *w*CI, *w*Ni1, and *w*Hm-c). As observed in the Oscar proteins of *w*Fur and *w*Sca (23), the Oscar homologs identified in this study encoded ankyrin repeats and the CifB C-terminus-like domains (papain-like cysteine proteinase domains; Fig. 2A and B). An Oscar homolog was also identified in the sequencing data derived from cells coinfected with *w*Fem and *w*CI. Although we could not reconstruct the genome of the *w*Fem strain due to technical difficulties in isolating *w*Fem from the coinfecting *w*CI, the Oscar homolog is presumed to be encoded by *w*Fem because the *w*CI strains used in this study did not carry Oscar homologs in their genomes (Fig. 2C and Table S1). Further diagnostic PCR confirmed that the Oscar homolog was consistently present in wild-caught *Eurema mandarina* butterflies infected with *w*Fem and *w*CI (*n* = 37) but not in those infected with *w*CI alone (*n* = 77), suggesting that the Oscar homolog is encoded by *w*Fem. Moreover, *w*Fem-infected females (*n* = 3) of *Eurema hecabe*, a close relative of *Eu. mandarina*, were Oscar positive.

**Table 2.** Genomic features of the Wolbachia strains used in this study.

Genome ID	*w*Kue	*w*CauA	*w*CauB	*w*Sca	*w*CI	*w*Ni1	*w*Hm-c	*w*Hm-t
Contigs	1	1	1	1	1	1	1	1
Genome size (bp)	1,305,037	1,713,404	1,703,391	1,322,867	1,358,360	1,410,166	1,492,082	1,542,158
G + C content (%)	35.3	35	34.2	33.7	34.1	34.1	34.0	34.1
CDS genes	1,276	1,595	1,632	1,258	1,249	1,335	1,347	1,426
Super group	A	A	B	B	B	B	B	B
rRNA	3	3	3	3	3	3	3	3
tRNA	34	34	34	34	33	34	34	34
Native hosts	*E. kuehniella*	*C. cautella*	*C. cautella*	*O. scapulalis*	*Eu. mandarina*	*T. ni*	*H. magnanima*	*H. magnanima*
Phenotype	CI	CI MK	CI	MK	CI	Non-MK Non-FM	Non-MK Non-FM Non-CI	MK
Sources	This study	This study	This study	This study	This study	This study	This study	Arai et al. (28)

MK, male killing; NMK, non-male killing; CI, cytoplasmic incompatibility.

**Fig. 3.**
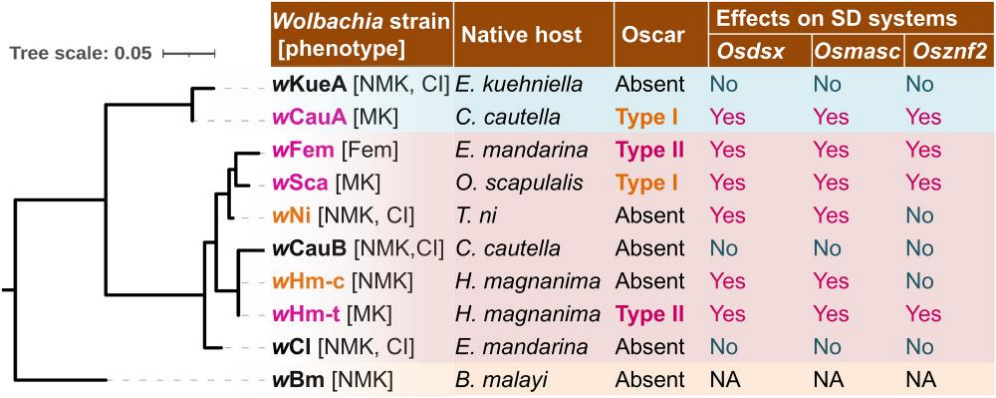
Phylogenies and phenotypes of *Wolbachia* strains and their effects on the sex-determination system in *Ostrinia* male cells. *wsp* and MLST genes were used to construct the phylogenetic tree. The classification of Oscar proteins was based on the phylogeny presented in Fig. 2C. The effects on splicing of each sex-determining gene analyzed in this study are presented as either Yes (feminized) or No (not affected). MK, male killing; NMK, nonmale killing; Fem, feminization; CI, cytoplasmic incompatibility; NA, not assessed.

To assess the phylogenetic associations of the Oscar homologs, we further searched Oscar homologs using the GenBank database, which identified Oscar-like proteins in four *Wolbachia* strains (*w*Gro in *Epagoge grotiana* [Tortricidae, Lepidoptera], *w*Spl in *Cydia splendana* [Tortricidae, Lepidoptera], *w*Fla in *Endotricha flammealis* [Pyralidae, Lepidoptera], and *w*Obs in *Cardiocondyla obscurior* [Formicidae, Hymenoptera]). Since Oscar proteins generally encoded CifB C-terminus-like domains, we constructed a phylogenetic tree of Oscar alongside CifB proteins found in the *Wolbachia* genomes we sequenced. Phylogenetic analysis classified the Oscar proteins into three clades: type I Oscar (Oscar [*w*Fur and *w*Sca], Ec-Oscar [*w*CauA], and Oscar-like proteins in *w*Gro [WP_265024289.1], *w*Spl [WP_264339188.1], and *w*Fla [WP_264954178.1]), type II Oscar (Hm-Oscar [*w*Hm-t] and Em/Eh-Oscar [*w*Fem]), and type III Oscar (*w*Obs [WP_174516118.1]) (Figs. 2C and S1). These Oscar homologs were highly diverse in sequences, and notably, Oscar in the newly sequenced *w*Sca strain was not identical to that in the previously sequenced *w*Sca strain (similarity: 90.4% in 1,797 amino acids; bit score: 3,241, BLASTp) and *w*Fur (similarity: 94.2% in 1,830 amino acids; bit score: 3,432) (45) because of deletions in ankyrin repeats (Table S1). Intriguingly, the type II Oscar homologs Hm-Oscar and Em-Oscar exhibited higher homology than the *w*Sca strains (similarity: 98.5% in 1,181 amino acids; bit score: 2,362). The MLST and genome-based phylogeny of the *Wolbachia* strains used in our analyses did not match the phylogeny of Oscar, suggesting horizontal transfer and/or rapid divergence of Oscar (Figs. 3 and S1).

### Oscar is absent in *w*Hm-c and *w*Ni that interact with insect sex-determination systems

The *w*Ni1 and *w*Hm-c strains, which induced female-type splicing of *masc* (*OsmascF*) and *dsx* (*OsdsxF*) in *Ostrinia* male cells, did not carry Oscar homologs (Fig. 3 and Table S1). In addition, comparative genomics did not identify any proteins that were exclusively present in *Wolbachia* strains that affected the host sex-determination system (*w*Hm-t, *w*Sca, *w*CauA, *w*Ni1, and *w*Hm-c) but not in other strains (*w*Kue, *w*CauB, and *w*CI; Fig. 2A). A protein cluster specific to *w*Hm-c and *w*Ni1 (i.e. absent in the other *Wolbachia* strains) consisted of three hypothetical proteins (63 amino acids: wHmcTK_11260, wNi_10160, and wNi_12630) that did not encode obvious domains and exhibited no homology to known MK genes (Tables S1 and S2). Genes carried by *w*Hm-c or *w*Ni1 that were present in some MK strains (*w*Sca, *w*CauA, and *w*Hm-t) but absent in non-MK strains (*w*CauB, *w*Kue, and *w*CI; Table S2) included a *wmk* homolog (wNi_11830), whereas others displayed extremely low or no homology to known MK genes (i.e. Oscar (23), Spaid (46), and PVMKp1 (47)).

## Discussion

Our cell transinfection assays proved effective in assessing the feminizing ability of *Wolbachia* strains. Comparative genomics further revealed the presence of Oscar homologs in these strains, which induced female-specific splicing of three sex-determining genes (*dsx*, *masc*, and *znf2*) in male *Ostrinia* cells. As previously argued (19, 27), the present study highlights a common mechanism underlying *Wolbachia*-induced MK and FM in lepidopteran insects: Oscar-induced suppression of Masc, leading to female-type sex determination and disruption of the dosage compensation system. Among the *Wolbachia* strains that feminized sex determination, *w*Sca was the most efficient at inducing the female-type isoforms of the *dsx* and *znf2* genes. This efficiency likely reflects its long-term adaptation to its native host, *O. scapulalis*, from which the OsM1 cell is derived. Notably, consistent with previous findings (25), the expression ratio of *dsx* (*OsdsxF* vs. *OsdsxM*) was lower than that of *masc* (*OsmascM* vs. *OsmascF*) and *znf2* (*Osznf2M* vs. *Osznf2F*) in the MK/FM *Wolbachia*-transinfected cells. These patterns may indicate a rapid effect of *Wolbachia* on the upstream sex determinants, with a subsequent time lag before influencing the expression levels of downstream factors.

Unexpectedly, our study also revealed that two Oscar-deficient *Wolbachia* strains (*w*Ni1 and *w*Hm-c) affect the sex-determination system of *Ostrinia*, albeit likely through a different mechanism from Oscar-bearing *Wolbachia* strains: Oscar-bearing *Wolbachia* strains (i.e. *w*CauA, *w*Sca, *w*Fem, and *w*Hm-t) affected *Osdsx*, *Osmasc*, and *Osznf2*, whereas Oscar-deficient *w*Ni1 and *w*Hm-c affected *Osdsx* and *Osmasc* but not *Osznf2*. These differences probably arose from the different machineries caused by different *Wolbachia* genes, i.e. *oscar* and other unknown genes. In *B. mori*, Znf2 is an upstream factor in the sex-determination cascade that regulates *dsx* sex-specific splicing (26). However, as the sex-determining gene cascade of *Ostrinia* (e.g. the hierarchical relationships among *Osmasc*, *Osznf2*, and *Osdsx*) is not fully understood, it remains unclear why only *Osznf2* is not affected by *w*Ni1 or *w*Hm-c. One possibility is that Oscar can suppress the functions of Znf2 (or the cascade containing Znf2) as well as Masc, whereas the factors carried by *w*Hm-c and *w*Ni1 cannot act on Znf2 or its upstream components. Our study illustrated that closely related bacteria in the genus *Wolbachia* can activate different functions to manipulate sex determination in a single insect species. Further investigation of the causes is warranted.

This study provides compelling evidence that *Wolbachia* strains (*w*CauA, *w*Ni1, and *w*Hm-c) that do not induce MK or FM in their native hosts (43, 44, 48) retain the inherent ability to manipulate the sex-determination systems of *Ostrinia*. Despite its ability to alter the sex-determination cascade in *Ostrinia* cells, *w*Hm-c does not affect the sex-determination cascade in the natural host *Homona magnanima* (22). These findings suggest that the abilities of *Wolbachia* to manipulate insect reproduction have been suppressed by the natural hosts but have been maintained through evolution. *Wolbachia*-induced reproductive manipulation can be influenced by the genetic background of the host (30–33). Under the condition of a female-biased sex ratio, mutations in the host genome that rescue males can be favored by selection. Indeed, the spread of suppressors of symbiont-induced MK has been observed in nature (18, 31, 37–40). For example, the spread of an MK suppressor in the butterfly *Hypolimnas bolina* was almost complete within 5 years (31, 36). In *Cadra cautella*, an MK phenotype, presumably induced by the Oscar-bearing *w*CauA, was recorded in the 1970s (49). In contrast *w*CauA did not induce MK in *C. cautella* collected around 2000 (48, 50), although it did induce MK when transferred to the closely related host *Ephestia kuehniella* (30). These findings suggest that *C. cautella* may have evolved a suppressor against *w*CauA-induced MK between the 1970s and 2000.

Since *w*Hm-c relatives are found in many species of tortrix moths (43, 44), it is possible that a suppressor against *w*Hm-c relatives evolved and became fixed in the ancestral species of tortrix moths. This suppressor cannot suppress the feminizing ability of Oscar-bearing *w*Hm-t, and thus, the mechanistic basis of this suppressor would be different from the suppressor of *C. cautella* against Oscar-bearing *w*CauA. Consequently, a new suppressor against *w*Hm-t may arise in *H. magnanima* in the future, leading to an evolutionary arms race between *Wolbachia* and the host. Recent studies have shown that MK microbes, including *Wolbachia*, have evolved diverse MK genes/mechanisms (22, 28, 46, 47). In response to these diverse MK mechanisms, host insects may have evolved various suppressors or suppression mechanisms, potentially masking the *Wolbachia* phenotypes.

In summary, our study demonstrates that the feminizing effect of *Wolbachia* strains on the sex-determining gene cascade is most likely the mechanistic basis of MK and FM in lepidopteran insects. This study highlights the effectiveness of combining cell culture systems and genomic analyses to uncover the inherent ability of *Wolbachia* to manipulate sex. These approaches could also prove effective in elucidating the mechanistic interplay between the host and other selfish reproductive manipulators. A molecular understanding of the commonality and diversity of microbial reproductive manipulations will contribute to a better understanding of the evolutionary interactions between selfish elements and their hosts.

## Materials and methods

### *Wolbachia* strains and insects

*Ephestia kuehniella* lines transinfected with *w*Kue, *w*CauA, and *w*CauB in each were maintained in the laboratory, as described by Sasaki et al. (30). Females infected with *w*CauA were mated with uninfected males (50). The insects were reared on a diet consisting of wheat bran, dried yeast, and glycerol (20:1:2) at 25 °C under a 16-h/8-h light/dark photoperiod. *Homona magnanima* infected with *w*Hm-t (Taoyuan, Taiwan (42)) or *w*Hm-c (Takao, Japan (28)) was maintained using Silkmate 2S (Nosan Co., Yokohama, Japan). The MK host line W^T12^ was maintained by crossing it with a *Wolbachia*-free normal sex ratio line, as described by Arai et al. (42). For *Eu. mandarina*, all-female blood coinfected with *w*Fem and *w*CI and a normal sex ratio line singly infected with *w*CI were collected on Tanegashima Island, Japan, and maintained on an artificial diet, as reported by Kageyama et al. (27). *Trichoplusia ni* harboring *w*Ni1 was collected in Matsudo, Chiba, Japan. The MK *w*Sca strain, maintained in BmM2 cells as described by Herran et al. (25), was used for the following assays.

### Transinfection of *Wolbachia* into cultured cells

Fat bodies septically isolated from *Wolbachia*-infected insects were placed in the cell lines AeAl2 (*w*CauA, *w*CauB, *w*Kue, *w*Ni1, *w*Hm-t, and *w*Hm-c) and BmN4 (*w*CI and *w*Fem). After confirming that *Wolbachia* was stably maintained by diagnostic PCR, *Wolbachia*-positive AeAl2/BmN4 cells were used as donors for *Wolbachia* transinfection into the *O. scapulalis* NARO-Ossc-M1 (OsM1) cell line. *Wolbachia*-infected AeAl2/BmN2 cells were passed through a 5.0-µm filter (cat. no. FJ25ASCCA050PL01, GVS, Via Roma, Italy), and six drops (∼300 µL) of the flow-through were added to the recipient OsM1 cell line.

### Reverse-transcription polymerase chain reaction

Total RNA (200–500 ng) extracted from harvested cells using Isogen II (Nippon Gene, Tokyo, Japan) or TRIzol RNA Isolation Reagent (Thermo Fisher Scientific, Waltham, MA, USA) was reverse-transcribed using a PrimeScript II 1st strand cDNA Synthesis Kit (Takara Bio, Shiga, Japan), according to the manufacturer's protocol. qPCR was performed with 5 µL of KOD SYBR (Toyobo), 0.4 µL each of the forward and reverse primers (10 pmol/µL), 2.2 µL of water, and 2.0 µL of the cDNA template. qPCR was performed in a LightCycler 96 System (Roche, Basel, Switzerland) with a temperature profile of 180 s at 95 °C; 40 cycles of 8 s at 98 °C, 10 s at 60 °C, and 10 s at 68 °C; and heating to 90 °C for melting curve analysis. The primers used in this study are listed in Table S3. The relative expression (*Osmasc*, *Osdsx*, and *Osznf2* vs. the control gene *Osef1a*) and the ratio of male-to-female splice variants were estimated.

### Genomic analyses

*w*Sca (in BmM2 cells, described by Herran et al. (25)), *w*Hm-c, *w*Ni1, *w*CauA, *w*Kue, *w*CI, and *w*Fem and *w*CI (i.e. coinfected into AeAl2 cells) were purified from cells harvested in 150 mL flasks, as described by Iturbe-Ormaetxe et al. (51) with several modifications. Cells were pelleted by centrifugation (900 × *g* for 5 min) and homogenized with glass beads using Multi-beads Shocker MB3000 at 2,000 rpm for 40 s (Yasui Kikai Co., Osaka, Japan). The lysates were passed through 5.0 and 1.2 µm filters and centrifuged at 15,000 × *g* for 30 min at 4 °C. Pellet-containing *Wolbachia* cells were subjected to DNA extraction using a NanoBind Big DNA Tissue kit (Circulomics, Baltimore, MD, USA) according to the manufacturer's protocol. The extracted DNA was sequenced using an Illumina short read (paired-end 150 bp) and MinION with a Rapid sequencing kit (Oxford Nanopore, Oxford, UK) with a Nanopore flange flow cell (R9.4) (Oxford Nanopore). For *w*CauB, DNA extracted from host insects harboring *Wolbachia* using a NanoBind Big DNA Tissue kit was sequenced using Illumina (paired-end 150 bp) and Nanopore platforms (ligation sequencing kit with Nanopore MinION flow cell [R10.4]; Oxford Nanopore). The Nanopore data were assembled using Flye v1.6 (52) and polished 3–5 times with Illumina data using Pilon (53) and minimap2 (54). The resulting circular *Wolbachia* genomes were annotated using DFAST (55). Protein homologies were analyzed using OrthoVenn 3 (https://orthovenn3.bioinfotoolkits.net). *Wolbachia cifB*, *wmk*, and *oscar* (accession numbers are presented in Fig. 2 and Table S1) (23, 29, 56) were used as queries to identify homologs from *Wolbachia* genomes using local BLASTn and BLASTp searches (default parameters). Motifs in *oscar* homologs were surveyed using InterPro (https://www.ebi.ac.uk/interpro/) and HHpred (https://toolkit.tuebingen.mpg.de/tools/hhpred). Oscar homologs were further analyzed using Blastn and Blastp searches against GenBank nr database.

### Phylogenetic analysis

MLST (*gatB*, *coxA*, *hcpA*, *ftsZ*, and *fbpA* genes) and *wsp* fragments from the *Wolbachia* strains used in this study were aligned using ClustalW (57) with a *Wolbachia* endosymbiont in *Brugia malayi* (*w*Bm) as an out-group. Single-copy genes (*n* = 638) conserved among *Wolbachia* strains (*w*Fur, *w*Sca, *w*Hm-t, *w*Hm-c, *w*Ni1, *w*CauA, *w*CauB, *w*CI, *w*Kue, *w*Gro, *w*Spl, *w*Fla, *w*Obs, and *w*Bm) were obtained using OrthoFinder (58) and concatenated, aligned, and trimmed using SeqKit (59), MAFFT (60), and trimAl (61), respectively. CifB and Oscar proteins were aligned using ClustalW (57), and trimmed using trimAl (61) in the default mode. These alignment files were used to construct a phylogenetic tree based on maximum likelihood with bootstrap resampling of 1,000 replicates using the IQTREE server (http://iqtree.cibiv.univie.ac.at/) and visualized using the iTOL web server (https://itol.embl.de/).

### PCR detection of *Em/Eh-oscar* gene

*Eurema mandarina* collected from Tanegashima Island (Kagoshima, Japan) (35) and *E. hecabe* collected from Kohama Island (Okinawa, Japan) were subjected to DNA extraction using a DNeasy kit (Qiagen, Hilden, Germany), according to the manufacturer's protocol. The DNA concentration was adjusted to 10 ng/µL and subjected to PCR using primer sets amplifying the complete sequences of *Em/Eh-oscar* and *Hm-oscar* (Table S3). *Em/Eh-oscar* was amplified using Emerald Amp Max Master Mix (TaKaRa) at 94 °C for 3 min; 35 cycles of 94 °C for 30 s, 62 °C for 30 s, and 72 °C for 3 min; and a final extension at 72 °C for 7 min, as described by Arai et al. (28).

### Statistical analysis

For RT-qPCR assays, we used the average cycle threshold value (Ct) for each sample and estimated the relative expression, as described by Herran et al. (25) and Sugimoto et al. (20). Elongation factor a (Ef1a) was used as the control gene. The 2^−ΔCt^ (i.e. Ct_Ave_ target gene −Ct_Ave_ ef1a) and 2^−ΔCtMal^2^−ΔCtFem^ (i.e. relative expression in males vs. females) values were calculated. The expression ratios of the splice variants (i.e. *DsxM/DsxF*, *MascM/MascF*, and *Znf2M/Znf2F*) were analyzed using the Steel–Dwass test in R software v4.0 (62).

## Supplementary Material

pgae348_Supplementary_Data

## Data Availability

The sequence read data are publicly available in DDBJ under the accession numbers PRJDB16497 (BioProject), DRA016973 (DDBJ Sequence Read Archive: DRA), and DRA017337 (DRA). *Wolbachia* genomes (contigs) are available in the DDBJ database under the following accession numbers: *w*CauA (SAMD00639214, AP028948), *w*CauB (SAMD00639215, AP028949), *w*Kue (SAMD00639216, AP028950), *w*CI (SAMD00639217, AP028951), *w*CI-Fem (SAMD00639218, AP028952), *w*Sca (SAMD00639219, AP028953), *w*Ni1 (SAMD00639220, AP028954), and *w*Hm-c (SAMD00654377, AP028994). Other data generated in this study are included in the manuscript and Supplementary material.

## Preprints

This manuscript was posted on a preprint: https://doi.org/10.1101/2024.02.12.579973
