# Supplementary material for: Cell-based assays and comparative genomics revealed the conserved and hidden effects of Wolbachia on insect sex determination
Source: PNAS Nexus. 2024 Aug 22;3(9):pgae348. doi: 10.1093/pnasnexus/pgae348 (PMC11370894; doi:10.1093/pnasnexus/pgae348)
Supplement: pgae348_Supplementary_Data [file pgae348_supplementary_data.zip › PNASNEXUS-PNASNEXUS-2024-00313R-s02.docx]

**Supplemental Figure**

**Cell-based assays and comparative genomics revealed the conserved and hidden effects of Wolbachia on insect sex determination**

Arai et al.,


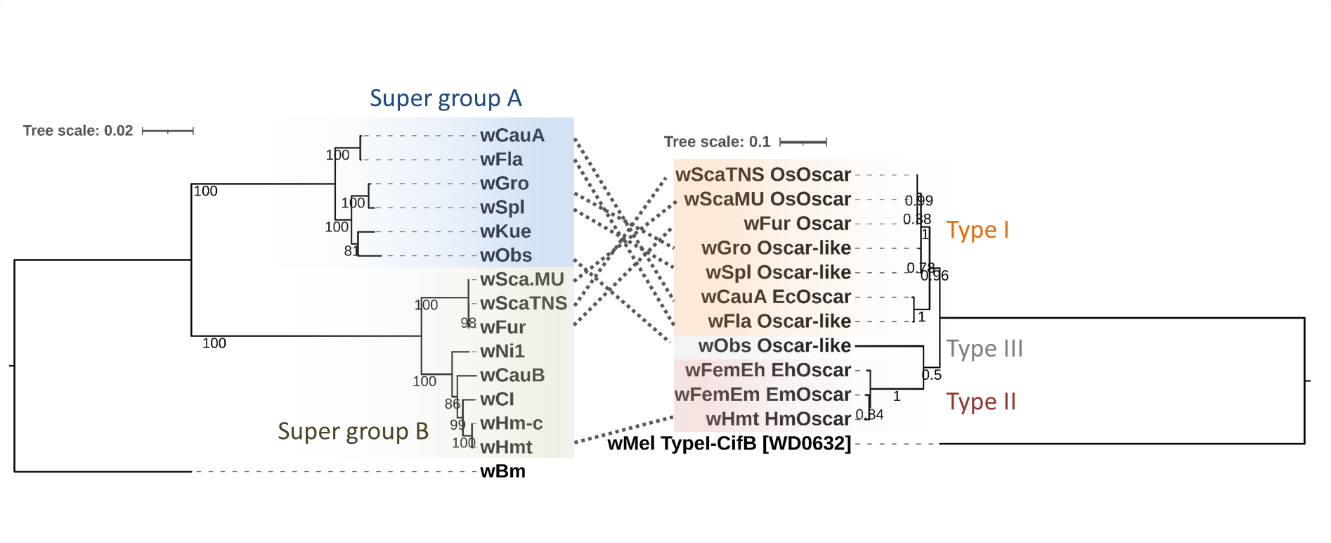


**Fig. S1.** Phylogenies of *Wolbachia* strains (left) and their Oscar homologs (right). Single-copy orthologous 658 proteins were used to construct the *Wolbachia* phylogenetic tree. *Wolbachia* supergroups are highlighted in blue (supergroup A) and beige (supergroup B). Oscar homologs were aligned with the CifB protein encoded by the *w*Mel strain, which was used to construct a phylogenetic tree. Oscar proteins are highlighted in orange (type I), red (type II), and grey (type III). The dashed lines indicate the position of the *Wolbachia* strains on the phylogenetic trees.
